# *ColourSpot*, a novel gamified tablet-based test for accurate diagnosis of color vision deficiency in young children

**DOI:** 10.3758/s13428-021-01622-5

**Published:** 2021-08-31

**Authors:** Teresa Tang, Leticia Álvaro, James Alvarez, John Maule, Alice Skelton, Anna Franklin, Jenny Bosten

**Affiliations:** 1 The Sussex Colour Group, School of Psychology, University of Sussex, Falmer, Brighton, BN1 9QH UK; 2 Facultad de Psicología, Universidad Complutense de Madrid, Pozuelo de Alarcón, Madrid 28883 Spain; 3 School of Psychology, University of Sussex, Falmer, Brighton, BN1 9QH UK; 4 Sussex Vision Lab, School of Psychology, University of Sussex, Falmer, Brighton, BN1 9QH UK

**Keywords:** Color vision deficiency, Visual development, Mobile health applications, Pediatric, Gamification

## Abstract

There is a need for a straightforward, accessible and accurate pediatric test for color vision deficiency (CVD). We present and evaluate *ColourSpot,* a self-administered, gamified and color calibrated tablet-based app, which diagnoses CVD from age 4. Children tap colored targets with saturations that are altered adaptively along the three dichromatic confusion lines. Two cohorts (Total, *N* = 772; Discovery, *N* = 236; Validation, *N* = 536) of 4–7-year-old boys were screened using the Ishihara test for Unlettered Persons and the Neitz Test of Color Vision. *ColourSpot* was evaluated by testing any child who made an error on the Ishihara Unlettered test alongside a randomly selected control group who made no errors. Psychometric functions were fit to the data and “threshold ratios” were calculated as the ratio of tritan to protan or deutan thresholds. Based on the threshold ratios derived using an optimal fitting procedure that best categorized children in the discovery cohort, *ColourSpot* showed a sensitivity of 1.00 and a specificity of 0.97 for classifying CVD against the Ishihara Unlettered in the independent validation cohort. *ColourSpot* was also able to categorize individuals with ambiguous results on the Ishihara Unlettered. Compared to the Ishihara Unlettered, the Neitz Test generated an unacceptably high level of false positives. *ColourSpot* is an accurate test for CVD, which could be used by anyone to diagnose CVD in children from the start of their education. *ColourSpot* could also have a wider impact: its interface could be adapted for measuring other aspects of children’s visual performance.

**Supplementary Information:**

The online version contains supplementary material available at 10.3758/s13428-021-01622-5.

## Introduction

Measuring individual visual performance in young children has long been a challenge (Robbins et al., 2003). This has meant that visual problems like color vision deficiency (CVD) or other visual abnormalities are often not detected as early as they could be. There is a need to develop widely accessible intuitive psychophysical methods targeted to young children.

Congenital CVD is the most common congenital visual disorder, affecting approximately 8% of males and 0.4% of females (Birch, 2012). In many countries there is no routine screening for CVD in schools or by optometrists (Atowa et al., 2019; Azizoǧlu et al., 2017; Ciner et al., 1999; Department of Health, 2009; Holroyd & Hall, 1997; Hopkins et al., 2013; Jadhav et al., 2017; Ramachandran et al., 2014; Stewart-Brown & Haslum, 1988; WHO Programme for the Prevention of Blindness and Deafness, 2003). One of the barriers to mass screening for CVD in children is a lack of widely accessible, high quality and child-appropriate diagnostic tests. However, identifying CVD in young children is necessary to support them in accessing an educational system that relies heavily on color.

Normal color vision involves the comparison of signals sent by three photoreceptor types: short (S), medium (M) and long (L) wavelength-sensitive cones. Anomalous trichromacy is the more common and milder form of red-green CVD, where all three cone types are present and functional, but there is an abnormality either in the L or in the M cone photopigment’s spectral sensitivity. Dichromacy is the more severe form of red-green CVD where there is an absence either of the L or of the M photopigment (Neitz & Neitz, 2000; Parry, 2015; Sharpe et al., 1999). It is important to detect CVD and identify how individuals can be supported, as it can significantly affect quality of life such as health, well-being and work (Barry et al., 2017; Chan et al., 2014; Cole, 2004, 2007; Cumberland et al., 2005; Steward & Cole, 1989; Tagarelli et al., 2004). Ninety percent of adults with CVD encounter problems in their daily lives (Steward & Cole, 1989), while children with CVD are at risk of social, behavioral and emotional difficulties, and adverse educational outcomes (Grassivaro Gallo et al., 1998, 2002; Suero et al., 2005; Thomas et al., 2018; Thuline, 1964). Educational materials, especially those for young children, such as reading schemes or mathematics activities (Birch, 2001), typically rely on color as a learning tool (Rinaldi et al., 2020; Suero et al., 2005), which makes them less accessible for children with CVD. In fact, one simulation of CVD suggests that 10% of tasks in educational textbooks are inaccessible for a child with CVD (Torrents et al., 2011). The dependence on color in education likely means that students with CVD are disadvantaged compared to their peers with normal color vision (Mehta et al., 2018).

Although there are a number of tests which have been used to detect CVD in children, they each have limitations. The gold standard diagnostic test in adults, the anomaloscope, has been used successfully with children older than 7, but the task of mixing a red and green to match a yellow is too demanding for younger children (Verriest, 1982). There are tests which aim to be child-friendly versions of other adult tests for CVD (e.g., the Ishihara test for Unlettered Persons), but their success varies greatly. Table 1 summarizes the properties of existing tests for CVD that have been designed for children or that are presented on tablet displays. An additional table summarizing tests for CVD aimed at adults which have also been used in children is included as Table S1 in the Supplementary Information (SI).

**Table 1:** A summary of tests for CVD either intended for use on children or presented on tablet displays

Test name	Adult sensitivity/specificity, numbers of adult test participants, and comparison test	Details of tests on children including numbers of participants and sensitivity/specificity where available	Recommended minimum age for test	Limitations
**Pseudoisochromatic**				
Ishihara for Unlettered Persons* (Ishihara & Ishihara, 1943)	0.98/1.00 (Birch & McKeever, 1993) (CVD=29, CVN=263) Ishihara, 1989 edition (Ishihara, 1989)	90% of 3–6-year-old children (*N*=40) successfully completed the test (Mäntyjärvi et al., 2000)	4 years (Birch & Platts, 1993)	†, ‡, § Requires shape knowledge and pathway tracing
Kojima-Matsubara Color Vision Test plates* (Matsubara & Kojima, 1957)	0.08/0.90 (Lee et al., 1997) (CVD=13, CVN=20) Anomaloscope	3–6-year-old children (*N*=40) successfully completed the test (Mäntyjärvi et al., 2000)	4 years (Mäntyjärvi et al., 2000)	†, ‡, § Requires animal knowledge
Pease Allen Color Test* (Pease & Allen, 1988)	0.87/1.00 (Pease & Allen, 1988) (CVD=23, CVN=210) Anomaloscope	97% of 3–6-year-old children passed the test (Pease & Allen, 1988)	3 years (Pease & Allen, 1988)	†, ‡
Color Vision Testing Made Easy (CVTME)* (Waggoner, 1994)	0.97/0.90 (Dain, 2010) (CVD=41, CVN=42) Anomaloscope	Children over 4 years successfully completed the test (Richardson et al., 2008)	3 years (Waggoner, 1994)	†, ‡, § Requires shape, animal and object knowledge
Neitz Test of Color Vision* (Neitz & Neitz, 2001)	1.00/0.86 (Block et al., 2004) (CVD=14, CVN=26) Anomaloscope	Tested in 4–12 years (*N*=115) and verified with genetic testing (Neitz & Neitz, 2001)	4 years (Neitz & Neitz, 2001)	†, ‡, § Requires shape knowledge
Color Vision Evaluation Test (CVET)* (Fish et al., 2020)	N/A	3–18 years 0.96/0.96 (Fish et al., 2020) (CVD=70, CVN=85) Ishihara 38-plate edition	3 years (Fish et al., 2020)	†, § Ability to identify orientations
**Oddity**				
Mollon-Reffin Minimalist Test* (Mollon et al., 1991)	N/A	3–10 years successfully completed the test (Shute & Westall, 2000). Children rated as most enjoyable test compared to CVTME, Neitz and Analphabetic Ishihara (Tekavčič Pompe & Stirn Kranjc, 2012)	3 years (Shute & Westall, 2000)	†, ‡
University of Waterloo Colored Dot Test* (Hovis et al., 2002)	0.57/1.00 (Hovis et al., 2002) (CVD=21, CVN=31) Anomaloscope	2.5–5 years 0.48/0.97 (Hovis et al., 2002) (CVD=25, CVN=524) Standard Pseudoisochromatic Plates (Ichikawa et al., 1979; Tanabe et al., 1978)	3 years (Hovis et al., 2002)	†
**Tablet-based**				
DoDo game* (Nguyen, Do, et al., 2014; Nguyen, Lu, et al., 2014)	N/A	6–17 years 0.81/1.00 (Nguyen, Do, et al., 2014) (CVD=16, CVN=16) Ishihara, 1998 edition (Ishihara, 1998)	2.5 years (Nguyen, Lu, et al., 2014)	†
Optopad (de Fez, Luque, Matea, et al., 2018)	0.75/0.94 (de Fez, Luque, Matea, et al., 2018) (CVD=16, CVN=50) Farnsworth Munsell 100-Hue (Farnsworth, 1943)	3–11 years 1.00/1.00 (de Fez, Luque, Matea, et al., 2018) (CVD=6, CVN=335) Ishihara, 2002 edition (Ishihara, 2002)	3 years (de Fez, Luque, Matea, et al., 2018)	†, § Ability to identify orientations of the Landolt C

The table outlines the test type, adult sensitivity and specificity values with their sample size and comparison test, rates of successful test completion in children with sensitivity and specificity if available, the recommended minimum age for test completion and the limitations of each test

***Note.*** **Definitions. Anomaloscope**: The anomaloscope is an optical instrument where individuals are asked to match different mixtures of red and green monochromatic lights to a yellow monochromatic light. It is the gold standard for assessing color vision; **CVD:** Participants with color vision deficiency (any CVD type, e.g., anomalous trichromacy, dichromacy); **CVN:** Participants with normal color vision; **N/A:** Not available; **Pseudoisochromatic tests**: These tests have an array of colored dots that form a figure (digits, pathways, letters, animals, or shapes) against an isoluminant background which individuals are asked to identify; **Oddity tests**: An odd-one-out task where individuals are asked to identify a colored target amongst distractors; **Sensitivity**: The rate at which a diagnostic test identifies true positives (i.e. individuals with a condition are correctly identified). For example, against the comparison test (in this example, the standard Ishihara test), the Ishihara Unlettered has a sensitivity of 0.98, indicating that 98% of individuals are correctly diagnosed as having a CVD (of any type) and 2% are false negatives (i.e., the Ishihara Unlettered diagnosed the individual as having normal color vision (CVN) but the standard Ishihara test diagnosed the same individual as CVD); **Specificity**: The rate at which a diagnostic test identifies true negatives (i.e. correctly identifies the absence of a condition). For example, when compared with the comparison test (in this example, the standard Ishihara test), the Ishihara Unlettered has a specificity of 1.00, indicating that 100% of CVN individuals were correctly categorized as CVN, and 0% of individuals were false positives (i.e., where the Ishihara Unlettered diagnosed the individual as CVD but the standard Ishihara test diagnosed the same individual as CVN).

Symbols. * The test was specifically designed for children. **† Inaccessibility.** The test is inaccessible for the public and/or requires specialized equipment and/or resources and/or a trained specialist administrator. **‡ Unknown validity**. The sensitivity and specificity values of the tests are unknown in children; **§ Unsuitability.** The test requires an understanding of numbers, orientation, shapes, and/or animals, or the task is so demanding that it is unsuitable for young children and children with additional educational needs.

Although Table 1 presents many options for diagnosing CVD in children, no existing test is suitable for mass-screening for CVD from as young as 4 years. Firstly, many of the tests require specialist equipment or resources and/or require a trained administrator. This requires screening of children for CVD to take place in an optometrist’s clinic or as part of a well-funded school CVD screening program. The World Health Organization does not currently recommend that color vision screening is included as part of population-based vision assessments (WHO Programme for the Prevention of Blindness and Deafness, 2003), and many countries do not always perform pediatric color vision screenings (Atowa et al., 2019), including Australia (Hopkins et al., 2013), India (Jadhav et al., 2017), Malaysia (Thomas et al., 2018), Turkey (Azizoǧlu et al., 2017), the United Kingdom (Department of Health, 2009) and 80% of states in the United States (Ciner et al., 1999). Secondly, although some tests have good sensitivity and specificity in adults, there is little evidence that they can diagnose CVD accurately in young children. Thirdly, many of the tests are not tailored to the capabilities of young children. For example, pseudoisochromatic plate tests may be difficult if children are not yet sufficiently familiar with the shapes, numbers and animals depicted on the plates (Tekavčič Pompe & Stirn Kranjc, 2012). Children may fail to identify these stimuli for reasons that are unrelated to their color vision. Many of the tests rely on the ability to integrate elements into a holistic percept, a skill which is known to be underdeveloped in young children (Kovács, 2000; Scherf et al., 2009). Some tests have complicated instructions, take too long to complete or are not sufficiently engaging for children’s limited attention and motivation.

One potential solution to the limited accessibility of pediatric tests for CVD is to use tablet-based methods. Such methods can provide mass testing at home, school and in the community, and are colorimetrically adequate for color vision testing if properly calibrated (Bodduluri, Boon, & Dain, 2017a; Dain & Almerdef, 2016; de Fez et al., 2016; de Fez, Luque, García-Domene, et al., 2018a). Two iPad-based tests for CVD in children, the DoDo game (Nguyen, Do, et al., 2014a; Nguyen, Lu, et al., 2014b) and the Optopad (de Fez, Luque, Matea, et al., 2018b), have been recently developed. Both tests only partially address the limitations of pediatric tests for CVD listed in Table 1. The DoDo game has the advantage of gamification (Abramov et al., 1984; Bodduluri, Boon, Ryan, & Dain, 2017b; Ling & Dain, 2018), but its calibration process has not been specified, which means that its effectiveness may vary between devices. Optopad is not gamified or specifically designed for children, and therefore may not sufficiently engage children’s motivation and attention, both factors that are known to influence children’s task performance (Diez et al., 2001; Taylor, 1970). Optopad also requires specialized software and hardware for calibration to be usable on different devices. Furthermore, Optopad identified only 6 out of 341 children as having CVD, a small sample to estimate the test’s sensitivity, and a surprisingly small prevalence rate (1.76% compared to an expected prevalence for their sample of 4.69%; Birch, 2012). This low measured prevalence along with low adult sensitivity and specificity (0.75/0.94) and the need to exclude some children (younger participants could enrol only after confirming that they were able to read numbers and correctly identify directions) means that Optopad’s child sensitivity and specificity values should be interpreted with caution. Both the DoDo game and Optopad are also not yet readily available for download, and there is nothing yet published to suggest that they are being used to diagnose CVD in children.

Here we present *ColourSpot,* a novel gamified tablet-based test for CVD in children from 4 years of age, which overcomes the limitations of existing pediatric tests for CVD. Our aim is for *ColourSpot* to be an accessible mass screening tool for CVD, usable by parents, teachers or optometrists in the home, classroom or clinic at the age that children start school. We combine rigorous psychophysics and color calibration with a child-friendly gamified and animated interface. Using an adaptive staircase procedure, *ColourSpot* measures discrimination thresholds for colored targets defined along the three (protan, deutan and tritan) dichromatic color confusion lines (the terms “protan,” “deutan” and “tritan” refer to types of CVD relating to the L-, M- and S-cone, respectively; protan, deutan and tritan confusion lines are lines in color space along which colors are not discriminable by dichromats missing the respective cone type). Thresholds are measured in an engaging and simple game where children tap the colored targets among luminance-defined distractors to reveal animated characters.

We outline the procedure and associated accuracy for calibrating display devices, and the psychophysics and design of *ColourSpot.* We present results evaluating *ColourSpot* in a two-stage design that ensures that its validation is based on data from a (validation) cohort independent of the (discovery) cohort used to identify optimal classification parameters for CVD versus normal color vision. Two hundred and thirty-six boys in the discovery cohort and 536 boys in the validation cohort completed the Ishihara test for Unlettered Persons (Ishihara Unlettered) and the Neitz Test of Color Vision (Neitz Test). We were interested in evaluating the latter since it has been proposed as a mass-screening tool for use in classrooms. Any child who made more than one error on the Ishihara Unlettered was tested on *ColourSpot*. A randomly selected control group of children who made no errors on the Ishihara Unlettered was also tested on *ColourSpot*. We present *ColourSpot’*s sensitivity and specificity against the Ishihara Unlettered as a pseudo gold standard.

## Methods

### Development of *ColourSpot*

#### Color calibration of iPad screens

**Fig. 1:**
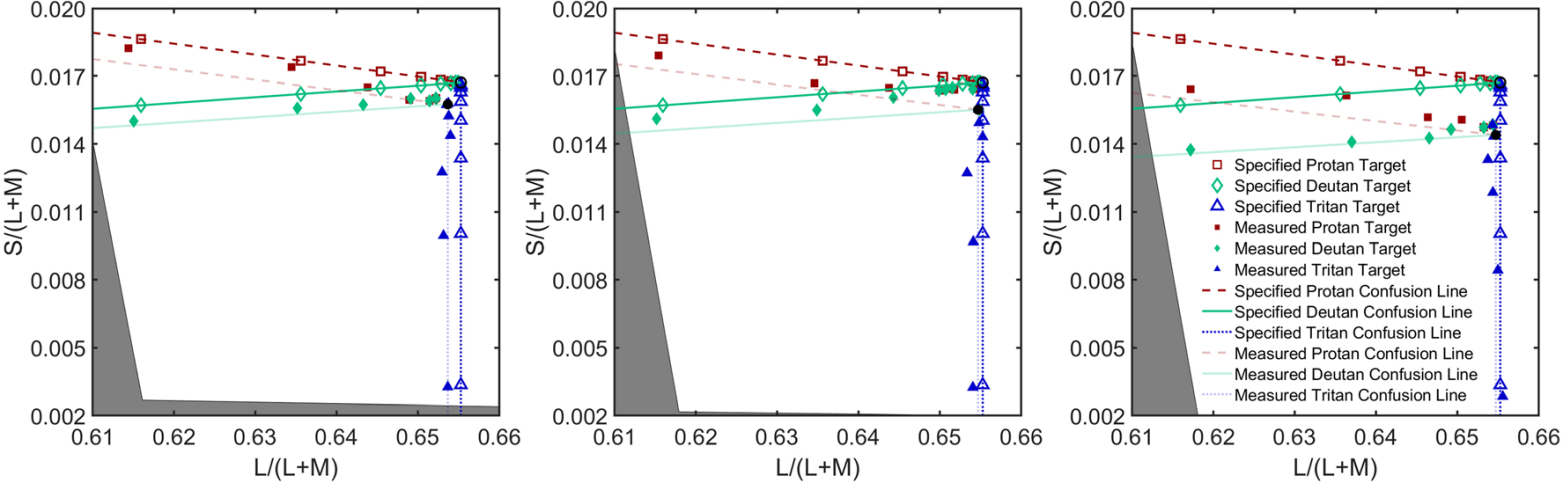
Calibration results for iPad Pro 9.7” (left), iPad (5th Generation) (middle) and iPad Air 2 (right) plotted in the MacLeod-Boynton chromaticity diagram (MacLeod & Boynton, 1979). The open symbols show the specified chromaticities and the closed symbols show the measured chromaticities. The red squares are targets on the protan confusion line (dashed line), the green diamonds are targets on the deutan confusion line (solid line) and the blue triangles are the targets on the tritan confusion line (dotted line). Each confusion line passes through the specified (open black circle) or measured (closed black circle) standard D65 white point. Opaque lines represent specified confusion lines (passing through the specified white point) and semi-transparent lines represent the confusion lines closest to the measured stimuli (passing through the measured white point). The solid grey area is outside the iPad’s gamut

In order to render colored stimuli accurately on different models of iPad which vary in their display of color, seven models of iPad (iPad 2, 2011; iPad (3rd generation), 2012; iPad (4th generation), 2012; iPad Air, 2013; iPad Air 2, 2014; iPad Pro 9.7,” 2016; and iPad (5th generation), 2017; Apple, Cupertino, CA, USA) were color calibrated. The gamma functions and the spectral power distributions of the display primaries were measured for a minimum of two units of each model using a PR655 Spectroradiometer (PhotoResearch, Chatsworth, CA, USA). Mean LMS to RGB transformation matrices were created using the radiance spectra of the three primaries, and mean gamma exponents were measured (and corrected). To check the quality of the calibration for each model, we presented our calibrated protan, deutan and tritan stimuli on each iPad model, and measured the spectra of the stimuli, calculating the LMS values of the presented stimuli from the spectra. Calibration was successful, showing relatively small residual systematic errors between the intended and measured chromaticities. The calibration errors tended to be systematic and therefore to place the stimuli on alternative but equally valid confusion lines (see Fig. 1). *ColourSpot* automatically detects the iPad model in use and provides the relevant iPad calibration for that model in order to achieve colorimetric accuracy for the test.

#### Luminance and tritan noise

There are large individual variations in spectral luminosity, particularly for individuals with CVD whose cone spectral sensitivities differ markedly from those of normal trichromats (Judd, 1945; Regan et al., 1994; Teller et al., 2003). To address this, we modeled the maximum luminance signal from our chromatic targets for dichromats and found this to be 10% of the background luminance. We therefore masked this potential signal by drawing the luminance of the targets and distractors from a linear distribution between ± 20% of the average luminance. Similarly, due to individual differences in cone fundamentals (Bosten, 2019) and small residual color calibration errors, binary tritan noise was added over the stimulus display at ± 16% of the background S/(L+M) value. This value was chosen after estimating the maximum available tritan signal from the protan and deutan targets (due either to individual differences in the peak sensitivities of the cone fundamentals or to calibration error) at 8%.

#### ColourSpot design

**Fig. 2:**
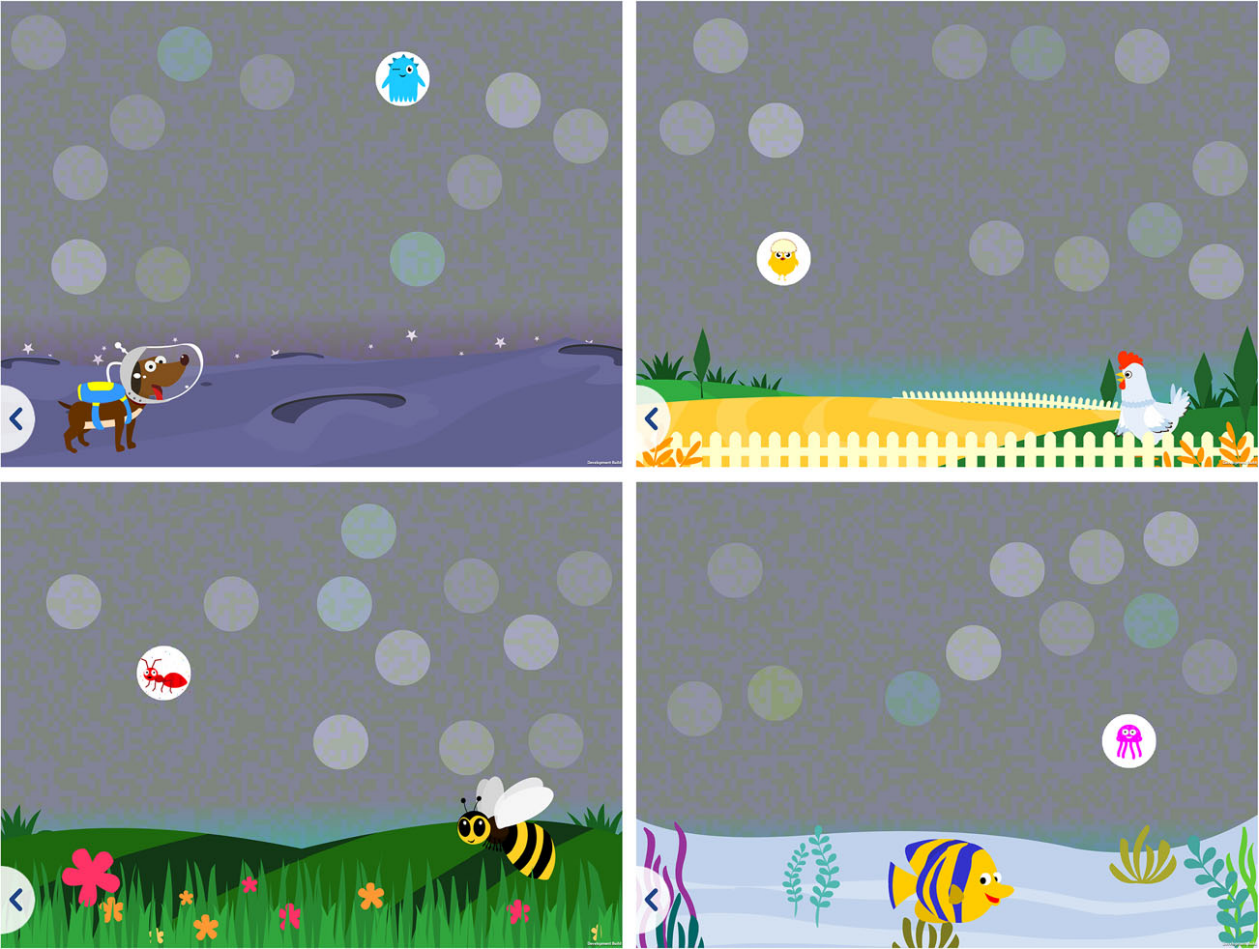
Examples of the various scenes showing an animation revealed when a target stimulus is tapped

**Fig. 3:**
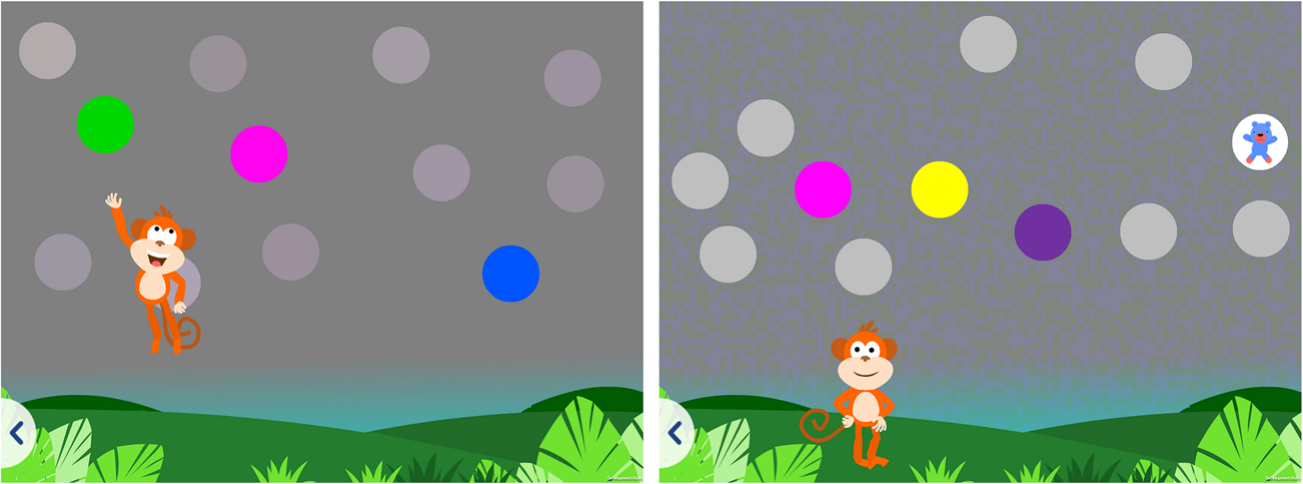
(Left) A demonstration tutorial where the monkey character demonstrates the rules of the game by tapping the highly saturated practice targets. **(**Right**)** A practice trial example where the cartoon animal animation is revealed from behind the colored practice target when the target is successfully tapped

*ColourSpot* measures protan, deutan and tritan discrimination thresholds. On each trial, participants are shown three targets selected along each of the dichromatic confusion axes (one protan, one deutan and one tritan target, see Fig. 1), and eight achromatic distractors varying in luminance (see Fig. 2). The test begins with a tutorial animation where an animated monkey character jumps up and touches a colored target whilst a child’s voiceover says, “tap a colored spot.” When a colored target is tapped it disappears to reveal a smaller animated character that moves and makes an appropriate animal sound (see Fig. 3). The tutorial uses highly saturated colored targets that are visible for both individuals with normal color vision and individuals with CVD. After the monkey demonstrates the rule of the game three times, the child begins practice trials. If the child taps a colored spot successfully five consecutive times in the practice trials, they progress automatically to the main part of the test which measures thresholds for the protan, deutan and tritan targets. On each trial, if a target is tapped, a cartoon animal animation along with an associated sound (e.g., a bird and a chirp) is revealed as a reward. There is no sound or animation if a distractor is tapped. Periodically, child voiceover clips are presented saying “Well done!,” “Amazing” and “You’re good at this!” as positive reinforcement for engaging with the game. During the main part of the test, there are five animated scene changes to keep the children engaged (Figs. 2 and 3 and supplementary video S1).

**Fig. 4:**
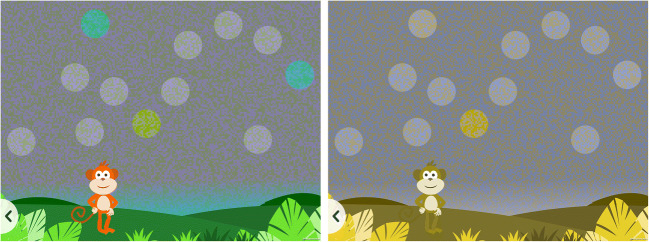
A simulation of how a deuteranope may perceive the game (right) compared to a normal trichromat (left). Please note that the simulation is for demonstration purposes only and perception may vary amongst CVD observers. The simulation was produced using Vischeck (Brettel et al., 1997; Lillo et al., 2014)

To measure thresholds, the colored targets in the main part of the test vary along the protan, deutan and tritan confusion lines according to an adaptive staircase procedure. Each target type (protan, deutan, tritan) has its individual staircase which begins at the highest available saturation level. If a target of a particular type is tapped, its saturation is multiplied by a factor of 0.5 for the next trial, thus decreasing the saturation of targets of that type, making detection of the target type more difficult. If a distractor is tapped, the saturations of all three target types are multiplied by a factor of 1.5 for the next trial, thus making the next trial easier. This design is intended to be helpful for children with severe CVD who may be performing at floor level for protan and deutan targets. Having a tritan target available should reduce their discouragement when they are unable to see the other targets (see Fig. 4 for an example trial simulated for a CVD observer). *ColourSpot* has two sequential sets of three staircases, with the first and then second set ending after the participant has reached 35 trials on each of the protan, deutan and tritan staircases. Once a staircase for a target type has reached 35 trials, the target type is no longer shown but is replaced by a distractor. Each trial remains on the screen until a target or distractor is tapped, and the locations of the targets and distractors are varied randomly on each trial. Children complete the test at their own pace, and at a moderate speed it can be completed in under 5 minutes. Thresholds are computed by fitting psychometric functions (see Results).

### Identifying and validating *ColourSpot*’s classification criteria for CVD

#### Participants

A total of 772 boys aged 4–7 years (mean 6.11 years, SD 0.90) were recruited from 27 primary schools in Sussex and London, UK. Each school provided information sheets and consent forms to parents inviting their children to participate in the research. Only participants with a completed and signed parent/guardian consent form were permitted to participate in the study. Participants were divided into two independent cohorts. The discovery cohort (N = 236, mean 6.17 years, SD 0.83) was used to identify the optimal criteria for assigning CVD versus normal color vision based on the data returned by *ColourSpot*. The validation cohort (N = 536, mean 6.09 years, SD 1.04) was used to test *ColourSpot*’s performance as a diagnostic test for CVD, applying the classification criteria identified using the discovery cohort’s data. We chose the sample size for the validation cohort using a custom bootstrap method which indicated that with a sample of 120 control children and 40 children with CVD, we would have 80% power to identify a 99% sensitivity within an 8% range and a 94% specificity within a 12% range. Based on a prevalence of 8%, a sample of 536 boys would be expected to include 43 boys with CVD. The values for sensitivity and specificity are based on those of the Ishihara test for adults since we were aiming to produce a similarly performing test for children. Table 2 provides a breakdown of the numbers of children of each age in the two cohorts.

**Table 2:** The numbers of participants of each age in the discovery cohort and in the validation cohort

Age (years)	Discovery Cohort	Validation Cohort
4	17	91
5	95	165
6	75	162
7	49	118
**Total**	**236**	**536**

The study adhered to the World Medical Association’s Declaration of Helsinki (2013), with the exception that it was not pre-registered. Ethical approval was granted by the University of Sussex Science & Technology Cross-Schools Research Ethics Committee (ER/TT283/2) and by the European Research Council Executive Agency.

#### Color vision tests

All children were administered the Ishihara Unlettered and the Neitz Test. The Ishihara Unlettered (Ishihara & Ishihara, 2016) contains a total of eight plates: three are example plates and five are test plates, including two plates that involve identifying a geometric shape and three plates that involve curve tracing. The Neitz Test is a multiple-choice pen and paper task that has one example plate and eight test plates. Each plate contains the outline of a geometric shape presented against a background of grey dots.

#### Procedure

Each participant was screened for CVD using the Ishihara Unlettered and, in order for us to compare *ColourSpot*’s results with those of another test designed for mass-screening, they also completed the Neitz Test. Any participant who made an error or traced irregularly on three or more plates of the Ishihara Unlettered was assigned to a “CVD” group. Any participant who made one or two errors or traced irregularly on the Ishihara Unlettered was assigned to an “inconclusive” group (see Fig. S1 in the Supplementary Information (SI) for the distribution of errors on the Ishihara Unlettered). Any child in the discovery cohort who generated inconclusive results on the Ishihara Unlettered was retested at a later date if possible (*n* = 6), and their group was reassigned if their results were conclusive at retest (*n* = 4 were reassigned to the control group). Children assigned to the inconclusive group in the validation cohort were not retested. A randomly selected subset of children who made no errors on the Ishihara Unlettered was assigned to a “control” group.

The children identified as CVD or inconclusive on the Ishihara Unlettered and the control sample were all tested on *ColourSpot*. Three iPad models (iPad Air 2, 2014; iPad Pro 9.7,” 2016; and iPad (5th generation), 2017) were used during testing (also see “Calibration” in the SI). To achieve sufficient trials to measure protan, deutan and tritan discrimination thresholds, participants had to complete a minimum of 40 trials. In the discovery cohort, one participant in the inconclusive group was unable to undertake *ColourSpot* due to school time restraints. This participant was unable to be retested at a later date as they had left the school. In the validation cohort, one participant in the CVD group who had additional educational needs did not want to play *ColourSpot*. Three participants (1 inconclusive, 2 controls) were excluded from the validation cohort as they had completed an insufficient number of trials. Owing to our selection criteria, the administrator of *ColourSpot* was aware of each child’s color vision diagnosis by the Ishihara Unlettered when the child was playing *ColourSpot*. However, *ColourSpot* is self-administered by the child with no interference by the administrator once it has started. Therefore, it would be impossible for the administrator to inadvertently bias the child’s performance selectively on one color confusion axis in a way that would influence the CVD diagnosis.

## Results

**Fig. 5:**
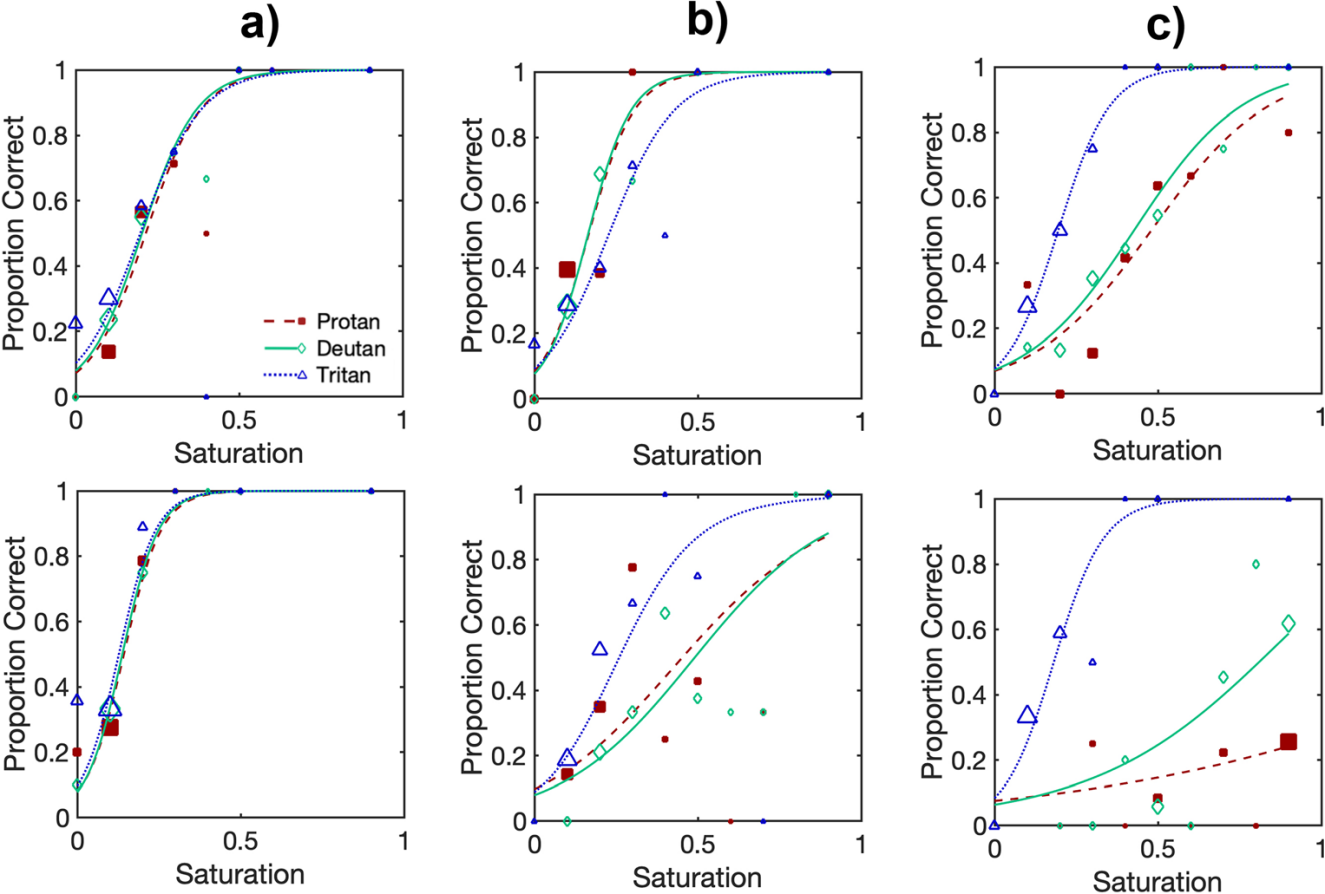
Examples of individual psychometric functions for six participants in the Control **(a)**, Inconclusive **(b)** and CVD **(c)** groups fit to their data from *ColourSpot*. Each participant has three psychometric functions representing their performance for detecting protan (red squares, dashed line), deutan (green diamonds, solid line) and tritan (blue triangles, dotted line) targets as a function of *ColourSpot*’s stimulus saturation. The size of the data points is proportional to an arbitrary power of the number of trials of each saturation. *ColourSpot’*s units are relative to the maximum possible saturation of a target on each confusion axis, but the greatest saturation tested on each axis was 0.9 times the maximum. In the chromaticity diagram (MacLeod & Boynton, 1979), the maximum available in gamut saturation (1.0) for protan targets had chromaticity coordinates L/(L+M) = 0.6160, S/(L+M) = 0.0186; the maximum in gamut saturation for deutan targets had chromaticity coordinates L/(L+M) = 0.6160, S/(L+M) = 0.0157; and the maximum in gamut saturation for tritan targets had chromaticity coordinates L/(L+M) = 0.6553, S/(L+M) = 0.0033

Data from the two staircases for each of the protan, deutan and tritan targets were combined and fit using non-parametric psychometric functions using local-linear fitting via the *modelfree* software package (Żychaluk & Foster, 2009). Figure 5 shows examples of individual psychometric functions from participants in the CVD, inconclusive and control groups. The figure shows that participants in the control group performed similarly for the protan, deutan and tritan targets, whereas participants categorized as CVD by the Ishihara Unlettered had higher thresholds for the protan and deutan targets compared to the tritan targets.

To find the optimal parameters of the model-free estimation of the psychometric function to achieve the best classification of CVD versus normal color vision (according to the Ishihara Unlettered) for the discovery cohort, we made a systematic search of parameter space. The optimal parameters were determined as having a fixed bandwidth of 0.70 for the model-free local linear fit, and a performance level of 0.21 (21%) on which to base threshold estimates (see Fig. S2 in the SI). For each participant and pair of fit parameters we found the minimum (most indicative of protan or deutan performance deficit) of the tritan:protan or tritan:deutan threshold ratio. Then we found the pair of fit parameters which maximized the distance between the CVD participant (according to the Ishihara Unlettered) with the largest ratio and the control participant with the smallest ratio. The model-free estimation was then implemented with the linear algebra library Armadillo (Sanderson & Curtin, 2016, 2018) to be made compatible with Apple software.

### Ability of *ColourSpot* to diagnose CVD

#### Discovery cohort

Existing diagnostic tests for CVD are typically based on a criterion score of raw protan and deutan thresholds between individuals (Barbur & Rodriguez-Carmona, 2015; Jurasevska et al., 2014; Mollon et al., 1991; Rabin et al., 2011). We found this to be a suboptimal metric on which to base classification for young children, because the raw thresholds along a given confusion axes do not account for individual differences in non-visual factors such as attention, motivation and engagement that influence task performance (Bosten et al., 2017; Dain & Ling, 2009; Ling & Dain, 2018; see Fig. S3 in the SI for a histogram of our raw protan and deutan thresholds; summary statistics for threshold ratios are provided in Table 4). Instead of raw thresholds, we used as a performance metric the minimum value between the ratio of tritan:protan or tritan:deutan thresholds. We propose this metric is effective at factoring out the influence of non-visual factors on task performance as they would affect thresholds along all three confusion axes equally. Given that congenital tritan deficiencies are extremely rare (Wright, 1952), the risk that a high tritan threshold would cancel high protan and deutan thresholds to create a high ratio in the presence of protan or deutan CVD is very low.

**Fig. 6:**
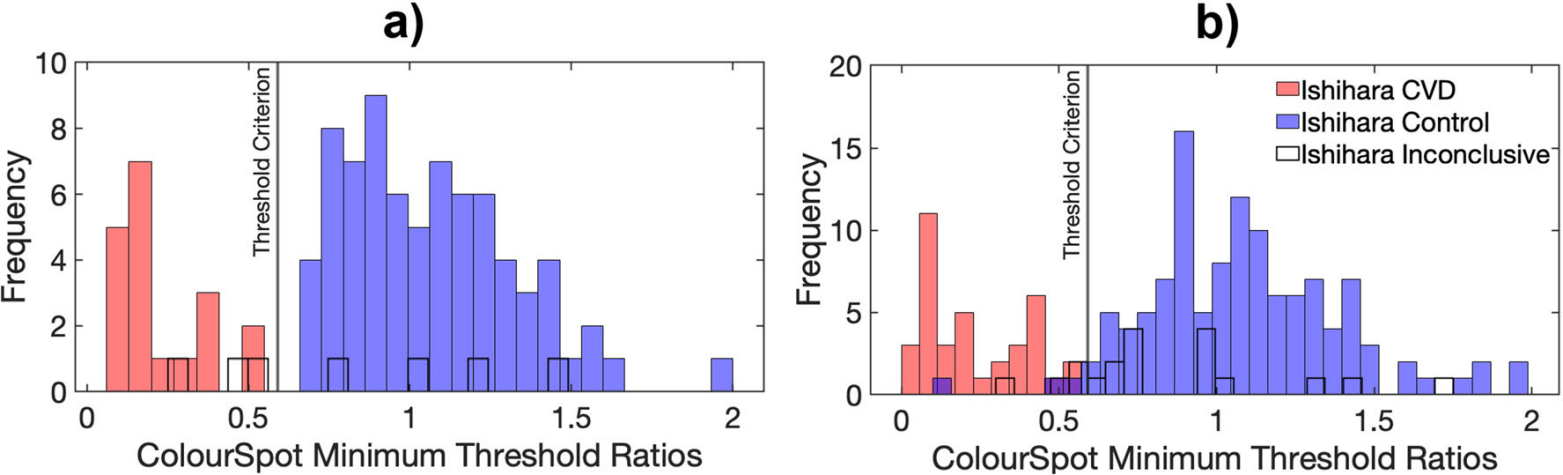
Histograms showing, for participants in the discovery cohort **(a)** and the validation cohort **(b)**, *ColourSpot*’s minimum threshold ratios of the tritan:protan or tritan:deutan thresholds for participants categorized as “CVD,” “Inconclusive” and “Control” by the Ishihara Unlettered. The solid vertical line is the criterion threshold ratio used to define the boundary between CVD and normal color vision

Figure 6a shows a histogram of the threshold ratios for the discovery cohort. There is a clear separation using this metric between groups who were classified either as CVD or as having normal color vision (Control) by performance on the Ishihara Unlettered. Results from the discovery cohort were used to decide on 0.59 as a criterion threshold ratio to define the boundary between a diagnosis of CVD and normal color vision. This was defined as halfway between the maximum threshold ratio for any participant in the CVD group and the minimum threshold ratio for any participant in the control group.

Using the Ishihara Unlettered as a pseudo gold standard test, *ColourSpot* has a nominal sensitivity of 1.00 and a specificity of 1.00 for the discovery cohort (see Table S2 for calculations). Results from *ColourSpot* suggest that the inconclusive group likely includes a combination of participants who have either mild CVD or normal color vision. Figure 6a shows that within the inconclusive group, four out of seven participants perform similarly on *ColourSpot* as participants in the control group (and thus likely have normal color vision). Three inconclusive participants’ threshold ratios lie within the distribution of threshold ratios for the CVD group (and thus likely have CVD).

#### Validation cohort

One hundred and seventy-four participants in the validation cohort played *ColourSpot* (Table 3). The optimal fit parameters and the criterion threshold ratio for defining the boundary between CVD and normal color vision from the results for the discovery cohort were applied without modification to the validation cohort (i.e., the classification method and criteria were independently applied to a new sample). Summary statistics for group threshold ratios for the validation cohort are provided in Table 4.

**Table 3:** Numbers of participants who played *ColourSpot* in the discovery and validation cohorts

Ishihara Unlettered groups	Discovery cohort	Validation cohort
CVD	19	37
Inconclusive	7	19
Control	74	118
Exclusions Untested	0 136	3 362
**Total**	**236**	**536**

Participants categorized as “CVD” made 3 or more errors on the Ishihara Unlettered. Participants categorized as “Inconclusive” made 1 or 2 errors on the Ishihara Unlettered. Participants categorized as “Control” were randomly selected from children who made no errors on the Ishihara Unlettered. Participants categorized as “Untested” are children who were categorized as having normal color vision by the Ishihara Unlettered but were not tested on *ColourSpot*

**Table 4:** The mean, standard error and 95% confidence intervals of the minimum tritan:protan or tritan:deutan threshold ratios in the discovery and validation cohorts

Cohort	Ishihara diagnosis	N	Mean	SE	95% Confidence intervals	
					Lower bound	Upper bound
Discovery	CVD	19	0.23	0.03	0.16	0.29
	Control	74	1.07	0.03	1.00	1.13
	Inconclusive	7	0.83	0.16	0.43	1.23
Validation	CVD	37	0.23	0.03	0.18	0.29
	Control	117	1.08	0.03	1.03	1.14
	Inconclusive	20	0.86	0.08	0.69	1.03

Again using the Ishihara Unlettered as a pseudo gold standard test, *ColourSpot* achieved a sensitivity of 1.00 and a specificity of 0.97 (see Table S3) for the independent validation cohort. If the criterion threshold ratio were applied to the performances of the inconclusive participants, 15 of the 19 inconclusive participants would be classified as having normal color vision, and one as having CVD. Three inconclusive participants were near the boundary between the normal and CVD groups and cannot be classified with confidence by either test. In the control group, three participants performed below the threshold criterion. Two of these were near the criterion threshold ratio and cannot be classified with confidence, but one participant in the control group performed similarly on *ColourSpot* to participants in the CVD group, and was likely given a false negative result by the Ishihara Unlettered (see Fig. S4 in the SI and Fig. 6b).

### Neitz Test of Color Vision

A summary of errors on the Neitz Test can be found in Fig. S5 and Fig. S6 of the SI. Briefly, we found that over 50% of boys made errors consistent with a CVD diagnosis. This implies, in agreement with other studies (Tekavčič Pompe, 2020; Tekavčič Pompe & Stirn Kranjc, 2012), that the Neitz Test does not provide an accurate diagnosis of CVD in young children.

## Discussion

Using gamification and psychophysical methods, *ColourSpot* gathers performance data that can be used to classify CVD similarly to or better than the Ishihara Unlettered. *ColourSpot* has many advantages relative to other pediatric tests for CVD and addresses the limitations of these tests. Firstly, our tritan control stimuli and associated “threshold ratio” measure allow us to distinguish visual from non-visual influences on task performance: If relatively poor performance is specific to the protan or deutan confusion lines, CVD is indicated, but if it applies to all three confusion lines, it is likely to be a result of non-visual factors like attention and task engagement. Secondly, gamification with a fun, intuitive and professionally animated interface helps children to maintain engagement and complete the test: 99% of children were able to complete *ColourSpot*, including 4-year-olds and children with additional educational needs. Thirdly, *ColourSpot* is accessible to anyone with an iPad, allowing it to be administered in any setting. Once downloaded, *ColourSpot* automatically provides the relevant calibration file for each iPad model. It is also self-administered, not requiring a trained administrator. This makes *ColourSpot* highly accessible around the world, either for home use with parents, at school with teachers, in the lab with researchers, or in the clinic with optometrists.

The technical features of *ColourSpot* distinguish it from other digitized tests for CVD. The combination of gamification and an adaptive staircase procedure is a psychophysical method well-suited to children. Particularly for children with CVD, the motivation offered by the game’s interface and the fact that stimulus contrast is adapted to current task performance both serve to maintain task engagement by reducing discouragement when they are unable to see targets. The addition of luminance and tritan noise allows us to guard against false negatives that might otherwise be caused by individual differences in color vision, particularly amongst individuals with CVD where cone spectral sensitivities vary significantly from the norm (Judd, 1945; Regan et al., 1994). *ColourSpot’*s tritan control stimuli allow our threshold ratio measure, which confers better classification accuracy for CVD versus normal color vision than using raw protan and deutan thresholds. Automatic application of model-specific color calibration allows mass testing and remote testing in different environments, which is an advantage against other digitized tests for CVD that are calibrated only for one particular device (Barbur & Rodriguez-Carmona, 2015; Rabin et al., 2011).

Our validation of *ColourSpot* using two independent cohorts provides statistical strength and gives confidence in the accuracy of classification. The classification algorithm optimized for the discovery cohort was applied to the validation cohort without modification, where it demonstrated similarly strong performance for classifying CVD versus normal color vision relative to the Ishihara Unlettered. For the validation cohort, the sensitivity (again against the Ishihara Unlettered) was 1.00 and the specificity 0.97. We chose the Ishihara Unlettered pragmatically as a pseudo gold standard because it is not possible to conduct the anomaloscope (the gold standard for adults) on our target age group, owing to its complex task demands. However, the sensitivity and specificity of the Ishihara Unlettered is itself imperfect, evidenced by the fact that it diagnosed 26 children as “inconclusive” in the current study. Using the Ishihara Unlettered as a pseudo gold standard in the current study may therefore lead us to underestimate the sensitivity and specificity of *ColourSpot* compared to using a fully accurate gold standard if diagnostic errors by the Ishihara Unlettered and *ColourSpot* are independent. Alternatively (and less likely), this may lead us to overestimate sensitivity and specificity for *ColourSpot* if diagnostic errors by *ColourSpot* are correlated with those of the Ishihara Unlettered (for example, if a certain type of mild CVD observer is missed by both tests). It is unclear from the Ishihara Unlettered test alone whether the 26 participants that scored inconclusively (1 or 2 errors) on the Ishihara Unlettered were anomalous trichromats or made errors on that test for reasons other than CVD (e.g., lapses in task engagement). However, four of the initially inconclusive participants in the discovery cohort were retested on the Ishihara Unlettered again within 2 months and made no errors, suggesting that they constituted false positive diagnoses by the Ishihara Unlettered. In contrast, *ColourSpot* provides a more secure assignment of color vision status for at least some of the inconclusive participants, suggesting that it may be more accurate at diagnosing CVD than the Ishihara Unlettered. In the validation cohort, one participant was diagnosed as normal by the Ishihara Unlettered but was classified as CVD by *ColourSpot*. Given the position of that participant’s threshold ratio near the centre of the CVD distribution (see Fig. 6b and Fig. S6 in the SI for the psychometric function of this participant), we believe that this is likely a false negative diagnosis by the Ishihara Unlettered, but a genetic test would be the only definitive way to confirm the diagnosis at the current age of this participant. As the protan and deutan confusion lines are so close to one another in color space, distinguishing protan CVD from deutan CVD is challenging. It is not possible to estimate *ColourSpot*’s accuracy for this classification using our current data, as our gold standard test (Ishihara Unlettered) does not accurately distinguish protan CVD and deutan CVD. We plan to explore *ColourSpot*’s ability to classify protan and deutan CVD types by testing it in adults and older children against the anomaloscope.

We aim that accurate diagnosis of CVD using *ColourSpot* from 4 years of age at the start of education will mitigate some of the negative impact of CVD on children’s education and well-being (Grassivaro Gallo et al., 1998, 2002; Mehta et al., 2018; Suero et al., 2005; Thomas et al., 2018; Thuline, 1964). The *ColourSpot* app provides advice sheets on CVD, designed by Colour Blind Awareness, a non-profit organization to raise awareness of CVD (http://colourblindawareness.org), which aims to help parents and teachers adopt strategies to mitigate some of the potential negative effects of CVD on children. Once generally released, *ColourSpot* can be used by parents, teachers and optometrists, and we hope that it will enable mass screening for CVD in young children due to its ease of use, psychophysical rigor and diagnostic accuracy.

*ColourSpot* is available to download for research purposes. The source code can be downloaded at https://osf.io/v5p2y/ and/or a fully-compiled version can be made available by contacting the senior authors. As well as the test itself, we also make available for research purposes the interface, animations and general methods which could be applied to measure other visual abilities in children.

## Supplementary Information

ESM 1 (DOCX 11.1 mb)
