# Supplementary material for: ColourSpot, a novel gamified tablet-based test for accurate diagnosis of color vision deficiency in young children
Source: Behav Res Methods. 2021 Aug 31;54(3):1148–60. doi: 10.3758/s13428-021-01622-5 (PMC9170621; doi:10.3758/s13428-021-01622-5)
Supplement: Supplementary file 1 — (DOCX 11.1 mb) [file 13428_2021_1622_MOESM1_ESM.docx]

**Accurate diagnosis of color vision deficiency in young children using *ColourSpot*, a new gamified tablet-based test**

**-Supplementary Information-**

**Table S1**

A summary of existing adult color vision tests which have been used in children, outlining the test type, adult sensitivity and specificity values with their sample size and comparison test, rates of successful test completion in children with sensitivity and specificity if available, the recommended minimum age for test completion and the limitations of each test

| **Test name** | **Adult sensitivity/ specificity, numbers of adult test participants and comparison test** | **Details of tests on children including number of participants and sensitivity/specificity where available** | **Recommended minimum age for test** | **Limitations** |
| --- | --- | --- | --- | --- |
| Anomaloscope  (Nagel, 1907) | 1.00/1.00  Gold standard | 7-year-olds able to complete the test (Jurasevska et al., 2014) | 7 years (Jurasevska et al., 2014; Verriest, 1982) | †, §, \|\|  Too demanding for young children |
| **Pseudo-isochromatic** | |  |  |  |
| Ishihara  (Ishihara, 1917) | 0.99 (Birch, 1997)/0.94 (Birch & McKeever, 1993)  (CVD=401(Birch, 1997), CVN=471(Birch & McKeever, 1993))  Anomaloscope (Birch, 1997)  Ishihara 1989 edition (Birch & McKeever, 1993; Ishihara, 1989) | 79–97% of 2–6-year-old children successfully completed the test (Choi & Hwang, 2009; Mäntyjärvi, 1991a) | 7 years (Verriest, 1982) | †, ‡, §  Requires number knowledge |
| Richmond Hardy-Rand-Rittler  (Hardy et al., 1954) | 1.00/0.98 (Cole et al., 2006)  (CVD=100, CVN=50)  Anomaloscope | N/A | 3 years (Hardy et al., 1954) | †, ‡, §  Requires shape knowledge |
| Standard Pseudo-isochromatic Plates  (Ichikawa et al., 1979; Tanabe et al., 1978) | 0.67/1.00 (Mäntyjärvi, 1987)  (CVD=21, CVN=205)  Anomaloscope | 6–9 years  (CVD=23, CVN=292)  0.87/0.99 (Haskett & Hovis, 1987)  Ishihara 1974 edition (Ishihara, 1974) | N/A | †, §  Requires number knowledge |
| Velhagen Pflügertrident Test  (Velhagen, 1980) | 0.61/1.00 (Mäntyjärvi, 1991b)  (CVD=31, CVN=394)  Anomaloscope | 71% of 2–6-year-old children successfully completed the test (Mäntyjärvi, 1991a) | 4 years (Birch & Platts, 1993) | †, ‡, §  Requires identification of a trident “E” figure orientated in different directions |
| KAMS  (Jurasevska et al., 2014) | N/A | 7–19 years  1.00/1.00 (Jurasevska et al., 2014)  (CVD=8, CVN= 265)  Anomaloscope | 7 years (Jurasevska et al., 2014) | †, §  Requires shape, symbol and number recognition |
| **Arrangement** | | | | |
| Farnsworth Munsell 100-Hue  (Farnsworth, 1943) | 1.00/0.83 (Seshadri et al., 2005)  (CVD=30, CVN=30)  Anomaloscope | N/A | 5 years (Kinnear & Sahraie, 2002) | †, ‡, \|\|  Performance is correlated with IQ (Cranwell et al., 2015) |
| Farnsworth Munsell D-15  (Linksz, 1966) | 0.59/0.98 (Oliphant & Hovis, 1998)  (CVD=70, CVN=81)  Anomaloscope | 8–15 years  0.92/0.99 (Shrestha & Shrestha, 2015)  (CVD=24, CVN=1090)  Ishihara | 5 years (Kinnear & Sahraie, 2002) | †  Performance is correlated with IQ (Cranwell et al., 2015) |
| Lanthony Desaturated D-15  (Lanthony, 1978) | 0.79/1.00 (Marechal et al., 2018)  (CVD=29, CVN=23)  Anomaloscope | N/A | 5 years (Lanthony, 1978) | †, ‡ |
| **Oddity** |  |  |  |  |
| City University Test  (Fletcher, 1984) | 0.95/0.83 (Oliphant & Hovis, 1998)  (CVD=70, CVN=81)  Anomaloscope | N/A | 4 years (Fanlo Zarazaga et al., 2019) | †, ‡, §  Requires knowledge of identifying orientation (up, down, left, right) verbally |
| **Computerized** |  |  |  |  |
| Cambridge Colour Test  (Regan et al., 1994) | 0.94/0.92 (Shinomori et al., 2016)  (CVD=32, CVN=162)  Anomaloscope | Modified version tested in 2–7 year old children (Goulart et al., 2008) | 7 years (Goulart et al., 2008; Ventura et al., 2002) | †, ‡, §  Identifying the orientation of the Landolt C stimulus by pressing corresponding keys on a keyboard |
| Rabin Cone Contrast Test  (Rabin et al., 2011) | 1.00/1.00 (Rabin et al., 2011)  (CVD=49, CVN=92)  Anomaloscope | N/A | N/A | †, ‡, §  Requires knowledge of letters |
| Colour Assessment and Diagnosis (CAD)  (Barbur & Rodriguez-Carmona, 2015) | 0.93/1.00 (Seshadri et al., 2005)  (CVD=30, CVN=30)  Anomaloscope | N/A | 4 years (Barbur & Rodriguez-Carmona, 2015) | †, ‡ |

***Note.* Definitions. Anomaloscope**: The anomaloscope is an optical instrument where individuals are asked to match different mixtures of red and green monochromatic light to different luminance levels of a yellow monochromatic light. It is the gold standard for assessing color vision; **CVD:** Participants with color vision deficiency (any CVD type, e.g. anomalous trichromacy, dichromacy); **CVN:** Participants with normal color vision; **N/A:** Not available; **Pseudo-isochromatic tests**: These tests have an array of colored dots that form a figure (digits, pathways, letters, animals or shapes) against an isoluminant background which individuals are asked to identify; **Arrangement tests**: Individuals are required to sort a set of colored stimuli by hue or saturation; **Oddity** **tests**: An odd-one-out task where individuals are asked to identify a colored target amongst other distractors; **Sensitivity**: The rate at which a diagnostic test identifies true positives (i.e. individuals with a condition are correctly identified). For example, in the table, against the comparison test (in this example, the anomaloscope), the Ishihara has a sensitivity of 0.99, indicating that 99% of individuals are correctly diagnosed as having a CVD (of any type) and 1% are false negatives (i.e., the Ishihara diagnosed the individual as having normal color vision (CVN) but the anomaloscope diagnosed the same individual as CVD); **Specificity**: The rate at which a diagnostic test identifies true negatives (i.e. correctly identifies individuals with absence of a condition). For example, in the above table, when compared with the comparison test (in this example, the Ishihara 1989 edition), the Ishihara test has a specificity of 0.94, indicating that 94% of individuals of CVN individuals were correctly categorized as CVN, and 6% of individuals were false positives (i.e., where the Ishihara Unlettered diagnosed the individual as CVD but the Ishihara diagnosed the same individual as CVN).

**Symbols.** **†** **Inaccessibility.** The test is inaccessible for the public and/or requires specialized equipment and/or resources and/or a trained specialist administrator. **‡** **Unknown validity**. The sensitivity and specificity values of the tests are unknown in children. **§** **Unsuitability.** The test requires an understanding of numbers, orientation, shapes, and/or animals or the task is so demanding that it is unsuitable for young children and children with additional educational needs. **||** **Technical Limitations.** The test design or instructions do not address general and non-visual task demands that could make it difficult for young children to complete, for example, test duration is longer than 5 minutes, no gamification and no adaptive staircase procedure.

**Distribution of errors on the Ishihara test for Unlettered Persons**

Figure S1 shows the distribution of errors (≥1) on the Ishihara Unlettered test for participants in the discovery and validation cohorts. The distribution for participants that have at least one error looks bimodal, with a peak in errors at 4, but a second peak at 1. Participants with 1 or 2 errors were assigned to an ‘Inconclusive’ group and retested at a later date whenever practicable (*n*=6).


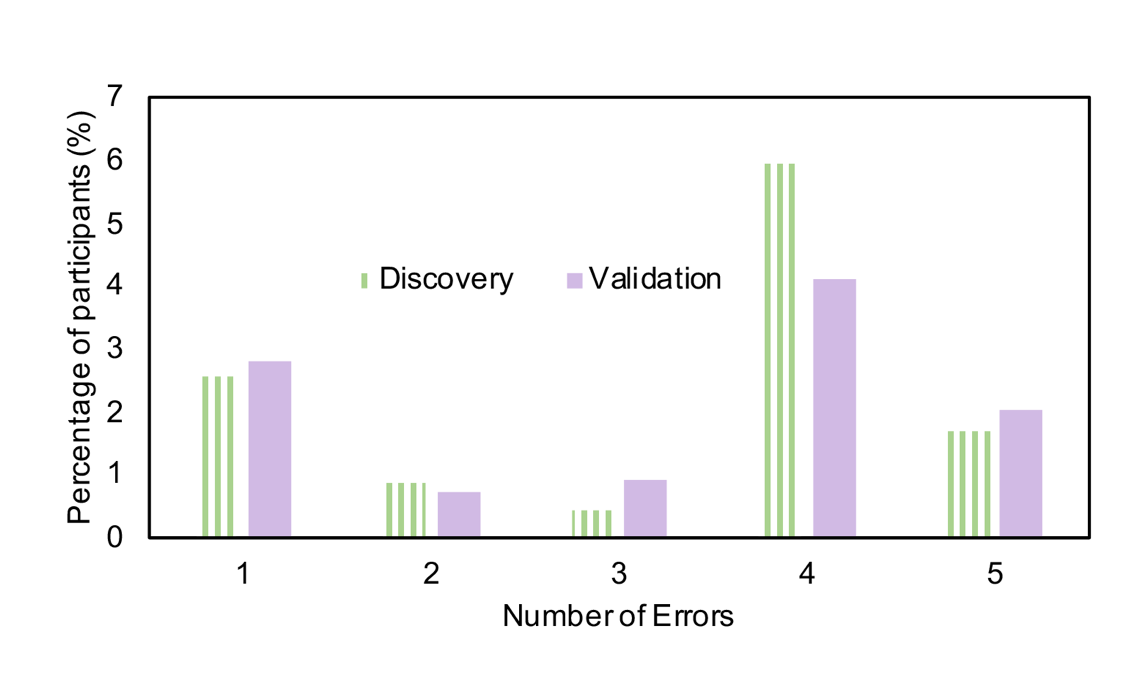


**Fig. S1** Percentages of participants who made errors on the Ishihara Unlettered test in the discovery cohort (*N*=236) and the validation cohort (*N*=536)

Of the six children in the discovery cohort retested, four children who made one or two errors on their first attempt later passed without error and were reassigned to the control group, and two children who again made one or two errors remained in the Inconclusive group.

**Calibration**

We investigated whether the protan, deutan and tritan thresholds measured from our participants differed between different iPad models. Mauchly’s test of sphericity indicated the assumption of sphericity had been violated, (*χ*^2^(2) = 0.54, *p* < 0.001), and therefore a Greenhouse-Geisser correction was used. A mixed ANOVA (within-subjects factors: color confusion axis (protan, deutan, tritan)); between-subjects factor: iPad models (iPad Air 2, 2014; iPad Pro 9.7”, 2016; iPad (5^th^ Generation), 2017) showed no significant interaction between the color confusion axes and iPad model (*F*_2.74, 371.16_ = 0.18, *p* = 0.90) on thresholds, suggesting that protan, deutan and tritan thresholds do not significantly differ between iPad models.

**Optimization of fit parameters for categorizing CVD versus normal color vision in the discovery cohort.**

We did a systematic search of the optimal bandwidth and percentage correct criterion for defining threshold, which maximized the distance in threshold ratio between the CVD and control groups for the discovery cohort (Fig. 5). The optimal parameters were determined as having a fixed bandwidth of 0.70 for the model-free (Żychaluk & Foster, 2009) local linear fit, and a performance level of 0.21 (21%) on which to base threshold estimates. Figure S2 shows the separation between groups in threshold ratio (Z) against the two parameters (X and Y).


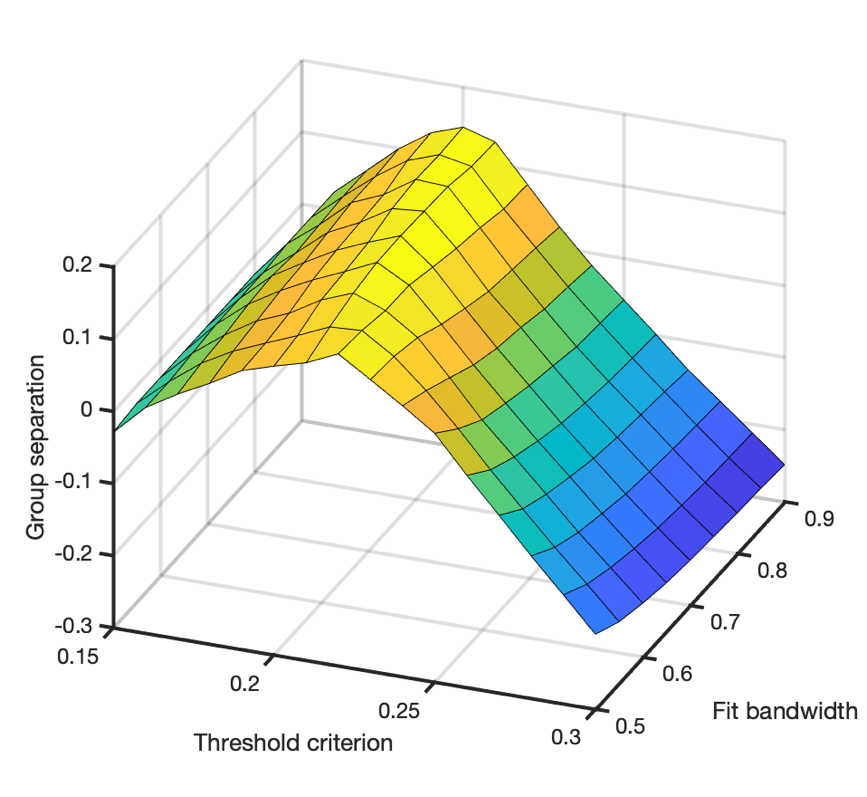


**Fig. S2** Results of a search for fit parameters that were optimal for distinguishing CVD from control participants. The Z-axis shows “separation” in threshold ratios between the participant in the CVD group with the largest threshold ratio and the participant in the control group with the smallest threshold ratio. The two fit parameters that were varied were the proportion correct at which threshold was defined (“Threshold criterion”) and the bandwidth (“Fit bandwidth”) for the local linear fit made using model-free (Żychaluk & Foster, 2009). The optimal parameters were defined at peak separation

**Raw protan and deutan thresholds**

*ColourSpot’s* tritan thresholds allowed us to quantify performance using the minimum ratio of tritan to protan thresholds or tritan to deutan thresholds. This offers an improvement in classification accuracy on many existing tests because non-visual influences on task performance (such as attention and task engagement) are factored out as they influence all three thresholds equally. The threshold ratio measure (Fig. 6) successfully separated those who were classified as CVD by the Ishihara Unlettered test and those who were classified on that test as having normal color vision into two different groups. By contrast, if raw thresholds were used, as is the case for many existing tests, classification accuracy was poorer (Fig. S3), since non-visual influences confer large individual differences in protan and deutan thresholds independently of color vision status.


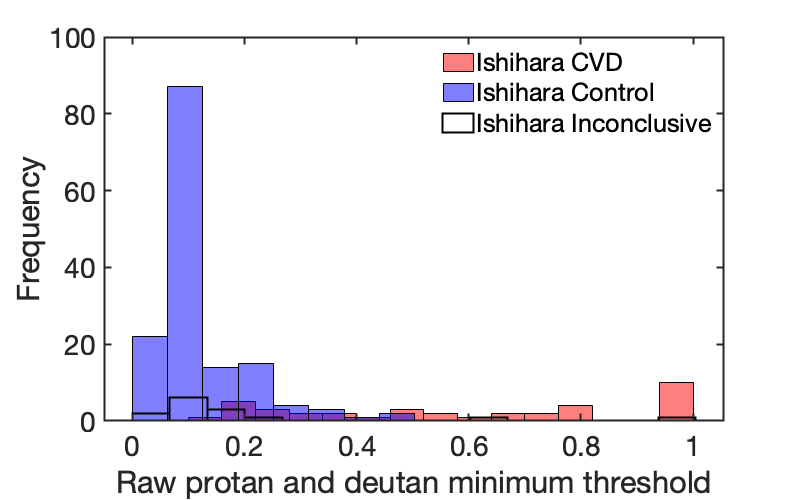


**Fig. S3** Histogram showing raw thresholds for participants in the discovery cohort grouped by color vision status assigned by the Ishihara Unlettered test. Plotted for each participant is the minimum of the protan or deutan threshold

***ColourSpot* Sensitivity and Specificity**

*Discovery Cohort*

Using the Ishihara Unlettered test as a pseudo gold standard, we identified a threshold ratio of 0.59 to be the classification criterion that distinguishes best between CVD and normal color vision, where a ratio smaller than 0.59 is indicative of a CVD. This threshold ratio was calculated by taking the average of the largest threshold ratio from the CVD group (0.52) and the smallest threshold ratio from the control group (0.66).

Excluding inconclusive participants, the criterion threshold ratio of 0.59 was applied to the discovery cohort (*N*=93) to estimate sensitivity and specificity values.

**Table S2**

Contingency table comparing the *ColourSpot* classification of CVD and normal color vision with that of the Ishihara Unlettered in the discovery cohort

| ***ColourSpot*** | **Ishihara test for Unlettered Persons** | |
| --- | --- | --- |
|  | **CVD** | **Normal** |
| **CVD** | 19 | 0 |
| **Normal** | 0 | 74 |
| **Total** | **19** | **74** |

Sensitivity = 19/19 = 1.00

Specificity = 74/74 = 1.00

*Validation Cohort*

For participants classified by the Ishihara Unlettered either as CVD or as having normal color vision (N = 155), the threshold ratio value of 0.59 was independently applied to the validation cohort to estimate sensitivity and specificity for *ColourSpot*.

**Table S3**

Contingency table comparing the *ColourSpot* classification of CVD and normal color vision with that of the Ishihara Unlettered in the validation cohort

| ***ColourSpot*** | **Ishihara test for Unlettered Persons** | |
| --- | --- | --- |
|  | **CVD** | **Normal** |
| **CVD** | 37 | 3 |
| **Normal** | 0 | 115 |
| **Total** | **37** | **118** |

Sensitivity= 37/37= 1.00

Specificity= 115/118= 0.97

For the validation cohort, *ColourSpot* classified three participants as having CVD which the Ishihara Unlettered had classified as having normal color vision. Two of these participants lie near the boundary between the normal and CVD group and cannot be classified with confidence. However, it appears that one of these participants (see Fig. S4 for their psychometric function) was likely a false negative diagnosis by the Ishihara Unlettered (classified as having normal color vision but in fact has CVD). Based on their performance on *ColourSpot*, we can see that this participant had much poorer performance for protan and deutan targets than tritan, suggesting that a CVD diagnosis is appropriate.


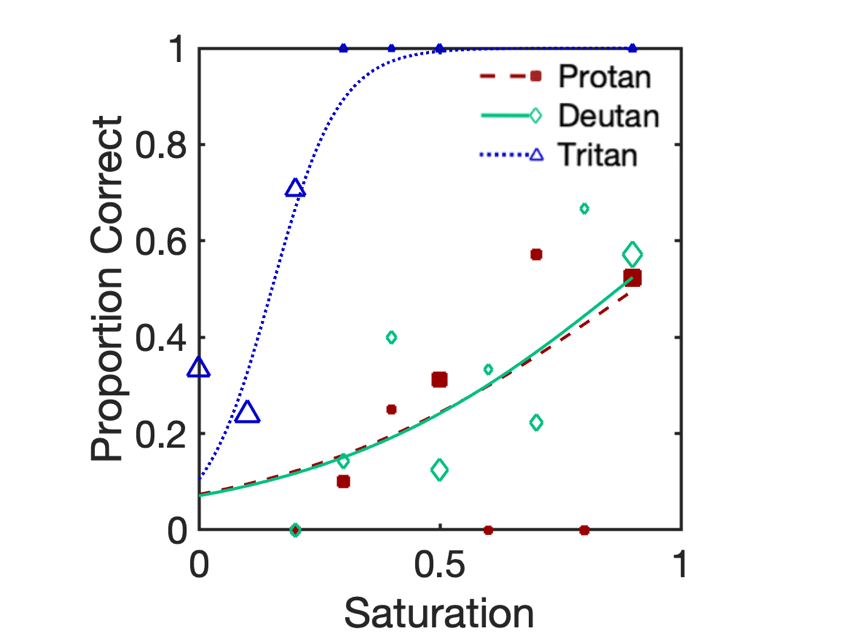


**Fig. S4** The psychometric functions of a participant in the validation cohort who was diagnosed as having normal color vision (made no errors) by the Ishihara Unlettered test but was diagnosed as CVD by *ColourSpot*

**Neitz Test of Color Vision**

Only 111 boys (47.0%) completed the Neitz Test without any errors in the discovery cohort and 246 (45.9%) in the validation cohort. The breakdown of errors by type is given in Fig. S5. The large number of errors is well above those expected from an 8% male prevalence for CVD, suggesting that the Neitz Test does not have good specificity for detecting CVD.


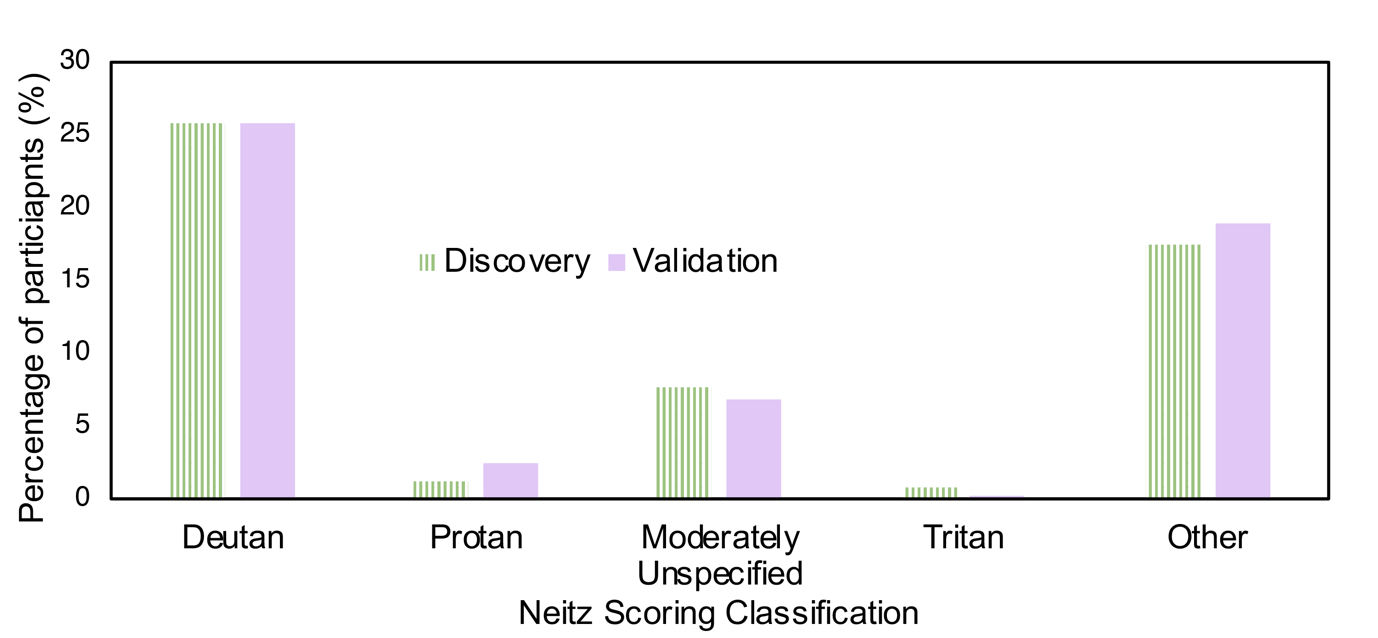


**Fig. S5** Percentage of participants that made errors of each type on the Neitz Test in the discovery and validation cohorts. “Moderately Unspecified” is where CVD errors were made but the classification of error type cannot be made, and “Other” is where errors were made that were not classified as a CVD error (e.g., an error due to misidentification of shape)

To further investigate the Neitz Test, the scoring classifications for the Neitz Test were compared to those of the Ishihara Unlettered test (Fig. S6). It can be seen that over 20% of children diagnosed as having normal color vision by the Ishihara Unlettered test (discovery and validation control groups) were classified as having deutan deficiencies by the Neitz Test. Additionally, about 17% of children who were diagnosed as having normal color vision by Ishihara Unlettered test made other errors on the Neitz Test. Furthermore, approximately 7% of children classified as CVD by the Ishihara Unlettered test were classified as having CVD that was “moderately unspecified” by the Neitz Test. These comparisons further suggest the Neitz Test over-diagnoses young children with CVD.
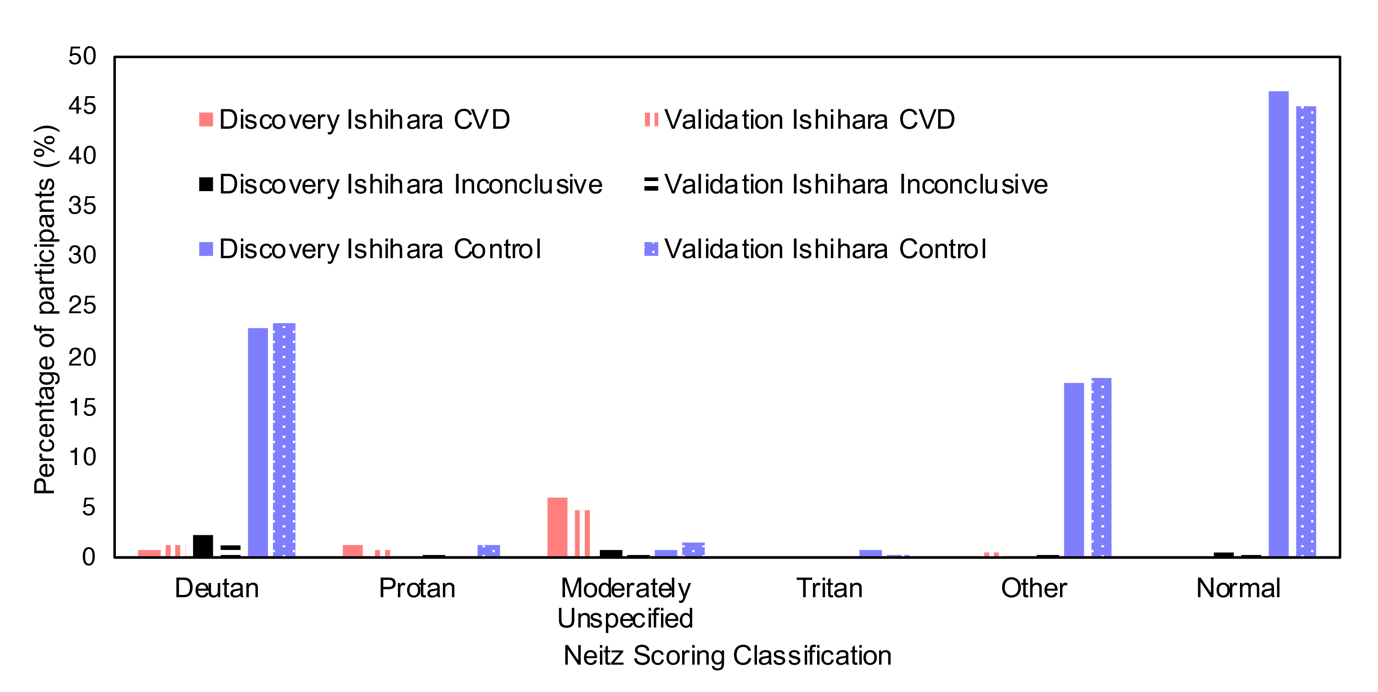


**Fig. S6** Scoring classifications on the Neitz Test against classifications made from the Ishihara Unlettered test in the discovery and validation cohorts

***References***

Barbur, J., & Rodriguez-Carmona, M. (2015). Color vision changes in normal aging. In A. J. Elliott, M. D. Fairchild, & A. Franklin (Eds.), *Handbook of Color Psychology* (pp. 180–196). Cambridge University Press. https://doi.org/10.1017/CBO9781107337930.009

Birch, J. (1997). Efficiency of the Ishihara test for identifying red-green colour deficiency. *Ophthalmic and Physiological Optics*, *17*(5), 403–408. https://doi.org/10.1046/j.1475-1313.1997.97000227.x

Birch, J., & McKeever, L. (1993). Survey of the accuracy of new pseudoisochromatic plates. *Ophthalmic & Physiological Optics : The Journal of the British College of Ophthalmic Opticians (Optometrists)*, *13*(1), 35–40. https://doi.org/10.1111/j.1475-1313.1993.tb00423.x

Birch, J., & Platts, C. (1993). Colour vision screening in children: an evaluation of three pseudoisochromatic tests. *Ophthalmic and Physiological Optics*, *13*(4), 344–349. https://doi.org/10.1111/j.1475-1313.1993.tb00489.x

Choi, S. Y., & Hwang, J. M. (2009). Ishihara test in 3- to 6-year-old children. *Japanese Journal of Ophthalmology*, *53*(5), 455–457. https://doi.org/10.1007/s10384-009-0716-1

Cole, B. L., Lian, K. Y., & Lakkis, C. (2006). The new Richmond HRR pseudoisochromatic test for colour vision is better than the Ishihara test. *Clinical and Experimental Optometry*, *89*(2), 73–80. https://doi.org/10.1111/j.1444-0938.2006.00015.x

Cranwell, M. B., Pearce, B., Loveridge, C., & Hurlbert, A. C. (2015). Performance on the Farnsworth-Munsell 100-hue test is significantly related to nonverbal IQ. *Investigative Ophthalmology and Visual Science*, *56*(5), 3171–3178. https://doi.org/10.1167/iovs.14-16094

Fanlo Zarazaga, A., Gutiérrez Vásquez, J., & Pueyo Royo, V. (2019). Review of the main colour vision clinical assessment tests. *Archivos de La Sociedad Espanola de Oftalmologia*, *94*(1), 25–32. https://doi.org/10.1016/j.oftal.2018.08.006

Farnsworth, D. (1943). The Farnsworth-Munsell 100-Hue and Dichotomous Tests for Color Vision. *Journal of the Optical Society of America*, *33*(10), 568–578. https://doi.org/10.1364/josa.33.000568

Fletcher, R. (1984). The City University Colour Vision Test. *Ophthalmic and Physiological Optics*, *4*(3), 279–279. https://doi.org/10.1111/j.1475-1313.1984.tb00367.x

Goulart, P. R. K., Bandeira, M. L., Tsubota, D., Oiwa, N. N., Costa, M. F., & Ventura, D. F. (2008). A computer-controlled color vision test for children based on the Cambridge Colour Test. *Visual Neuroscience*, *25*(3), 445–450. https://doi.org/10.1017/S0952523808080589

Hardy, L. H., Rand, G., & Rittler, M. C. (1954). H–R–R Polychromatic Plates. *Journal of the Optical Society of America*, *44*(7), 509–523. https://doi.org/10.1364/josa.44.000509

Haskett, M. K., & Hovis, J. K. (1987). Comparison of the standard pseudoisochromatic plates to the Ishihara color vision test. *Optometry and Vision Science*, *64*(3), 211–216. https://doi.org/10.1097/00006324-198703000-00008

Ichikawa, H., Hukami, K., Tanabe, S., & Kawakami, G. (1979). Standard Pseudoisochromatic Plates Part 2. *Clinical and Experimental Optometry*, *62*, 362. https://doi.org/10.1111/j.1444-0938.1979.tb02331.x

Ishihara, S. (1917). *Tests for color-blindness*. Handaya.

Ishihara, S. (1974). *Tests for Colour-Blindness*. Kanehara Shuppan.

Ishihara, S. (1989). *The series of plates designed as a test for colour-deficiency*. Kanehara.

Jurasevska, K., Ozolinsh, M., Fomins, S., Gutmane, A., Zutere, B., Pausus, A., & Karitans, V. (2014). Color-discrimination threshold determination using pseudoisochromatic test plates. *Frontiers in Psychology*, *5*, 1–7. https://doi.org/10.3389/fpsyg.2014.01376

Kinnear, P. R., & Sahraie, A. (2002). New Farnsworth-Munsell 100 hue test norms of normal observers for each year of age 5-22 and for age decades 30-70. *British Journal of Ophthalmology*, *86*(12), 1408–1411. https://doi.org/10.1136/bjo.86.12.1408

Lanthony, P. (1978). The desaturated panel D-15. *Documenta Ophthalmologica*, *46*(1), 185–189. https://doi.org/10.1007/BF00174107

Linksz, A. (1966). The Farnsworth panel D-15 test. *American Journal of Ophthalmology*, *62*(1), 27–37. https://doi.org/10.1016/0002-9394(66)91673-4

Mäntyjärvi, M. (1987). An Evaluation of the Standard Pseudoisochromatic Plates (SPP 1) in Clinical Use. In G. Verriest (Ed.), *Colour Vision Deficiencies VIII. Documenta Ophthalmologica Proceedings Series* (pp. 125–131). Springer. https://doi.org/10.1007/978-94-009-4275-2_18

Mäntyjärvi, M. (1991a). Colour Vision Testing in Pre-School-Aged Children. *Ophthalmologica*, *202*(3), 147–151. https://doi.org/10.1159/000310196

Mäntyjärvi, M. (1991b). Velhagen Pflügertrident pseudoisochromatic plates in screening congenital red-green vision defects. *Graefe’s Archive for Clinical and Experimental Ophthalmology*, *229*(2), 145–146. https://doi.org/10.1007/bf00170546

Marechal, M., Delbarre, M., Tesson, J., Lacambre, C., Lefebvre, H., & Froussart-Maille, F. (2018). Color vision tests in pilots’ medical assessments. *Aerospace Medicine and Human Performance*, *89*(8), 737–743. https://doi.org/10.3357/AMHP.5009.2018

Nagel, W. A. (1907). I. Zwei Apparate für die augenärztliche Funktionsprüfung. *Ophthalmologica*, *17*(3), 201–222. https://doi.org/10.1159/000291204

Oliphant, D., & Hovis, J. K. (1998). Comparison of the D-15 and City University (second) color vision tests. *Vision Research*, *38*(21), 3461–3465. https://doi.org/10.1016/S0042-6989(98)00117-5

Rabin, J., Gooch, J., & Ivan, D. (2011). Rapid quantification of color vision: The cone contrast test. *Investigative Ophthalmology and Visual Science*, *52*(2), 816–820. https://doi.org/10.1167/iovs.10-6283

Regan, B. C., Reffin, J. P., & Mollon, J. D. (1994). Luminance noise and the rapid determination of discrimination ellipses in colour deficiency. *Vision Research*, *34*(10), 1279–1299. https://doi.org/10.1016/0042-6989(94)90203-8

Seshadri, J., Christensen, J., Lakshminarayanan, V., & Bassi, C. J. (2005). Evaluation of the new web-based “Colour Assessment and Diagnosis” test. *Optometry and Vision Science*, *82*(10), 882–885. https://doi.org/10.1097/01.opx.0000182211.48498.4e

Shinomori, K., Panorgias, A., & Werner, J. S. (2016). Discrimination thresholds of normal and anomalous trichromats: Model of senescent changes in ocular media density on the Cambridge Colour Test. *Journal of the Optical Society of America A*, *33*(3), A65-76. https://doi.org/10.1364/josaa.33.000a65

Shrestha, R. K., & Shrestha, G. S. (2015). Assessment of color vision among school children: A comparative study between the Ishihara test and the Farnsworth D-15 test. *Journal of the Nepal Medical Association*, *53*(200), 266–269. https://doi.org/10.31729/jnma.2743

Tanabe, S., Ichikawa, H., Hukami, K., & Kawakami, G. (1978). New pseudoisochromatic plates for congenital color vision defects. *Japanese Journal of Clinical Ophthalmology*, *32*(3), 479–487.

Velhagen, K. (1980). *Pflügerhaken-Tafeln zur Prüfung des Farbensinnes*. Thieme, Leipzig.

Ventura, D., Rodrigues, A., Moura, A., Vargas, A., Costa, M., de Souza, J., & Silveira, L. (2002). Color Discrimination Measured by the Cambridge Colour Vision Test (CCVT) in Children and Adults. *Investigative Ophthalmology & Visual Science*, *43*(13), 3796.

Verriest, G. (1982). Colour vision tests in children. *Documenta Ophthalmologica Proceedings Series*, *33*, 175–178.

Żychaluk, K., & Foster, D. H. (2009). Model-free estimation of the psychometric function. *Attention, Perception, and Psychophysics*, *71*(6), 1414–1425. https://doi.org/10.3758/APP.71.6.1414
